# Polyimide-coated carbon electrodes combined with redox mediators for superior Li-O_2_ cells with excellent cycling performance and decreased overpotential

**DOI:** 10.1038/srep42617

**Published:** 2017-02-15

**Authors:** Seon Hye Yoon, Yong Joon Park

**Affiliations:** 1 Department of Advanced Materials Engineering, Kyonggi University, 154-42 Gwanggyosan-ro, Yeongtong-gu, Suwon-si, Gyeonggi-Do, 443-760, Korea

## Abstract

We report an air electrode employing polyimide-coated carbon nanotubes (CNTs) combined with a redox mediator for Li-O_2_ cells with enhanced electrochemical performance. The polyimide coating on the carbon surface suppresses unwanted side reactions, which decreases the amount of accumulated reaction products on the surface of the air electrode during cycling. The redox mediators lower the overpotential of the Li-O_2_ cells because they can easily transfer electrons from the electrode to the reaction products. The low overpotential can also decrease the side reactions that activate at a high potential range. Specifically, the CsI redox mediator effectively interrupted dendrite growth on the Li anode during cycling due to the shielding effect of its Cs^+^ ions and acted as a redox mediator due to its I^−^ ions. LiNO_3_ also facilitates the decrease in side reactions and the stabilization of the Li anode. The synergic effect of the polyimide coating and the electrolyte containing the LiNO_3_/CsI redox mediator leads to a low overpotential and excellent cycling performance (over 250 cycles with a capacity of 1,500 mAh·g_electrode_^−1^).

Until now, most road transportation systems have relied on internal combustion engines using petroleum-based fuels. However, with increasing environmental concerns around the world, new transportation systems based on electric motors and batteries, such as electric vehicles (EVs), have attracted significant interest due to their remarkable decrease in the production of CO_2_. The major technical hurdle for the wide commercialization of EVs is that present-day batteries cannot satisfy the energy-storage capacity requirements for pure EVs. Therefore, a great deal of studies has been devoted to developing new novel battery systems with superior energy densities^1^,^2^,^3^,^4^,^5^,^6^,^7^. Nonaqueous Li-O_2_ batteries are the most impressive next-generation battery systems, because their energy-storage density far exceeds that achievable with all other battery chemistries^8^,^9^,^10^,^11^,^12^,^13^,^14^,^15^,^16^. However, they face several major challenges, including high overpotentials and limited cycling performance^17^,^18^,^19^,^20^,^21^. These challenges are inherently linked to the fundamental nature of Li-O_2_ cells such as the high reactivity of reduced oxygen species formed during the discharge process, electrolyte instability during the oxygen reduction and oxygen evolution reactions, and the difficulty of perfectly dissociating non-conductive reaction products^22^,^23^,^24^,^25^,^26^,^27^,^28^,^29^,^30^.

The reaction mechanism of nonaqueous Li-O_2_ batteries at the porous air electrode surface is based on the formation and decomposition of solid reaction products such as Li_2_O_2_. During the discharge process (oxygen reduction reaction), molecular oxygen is reduced to superoxide and combined with lithium ions to form reaction products. However, these oxygen species lead to the decomposition of the electrolyte as a side effect. Moreover, during the charging process (oxygen evolution reaction), the reaction product Li_2_O_2_ reacts with carbon (a base material of air electrodes) and forms Li_2_CO_3_ (an unwanted reaction product). In addition, the electrolyte is also oxidized and decomposed at a high potential range during charging. These side reactions deplete the electrolyte solution during cycling and result in the accumulation of unwanted reaction products such as Li_2_CO_3_ and organic materials from the decomposition of electrolyte on the surface of the porous air electrode. Residual unwanted reaction products can clog the air electrode and limit the cycling performance of Li-O_2_ cells. Notably, carbon in the air electrode activates the side reactions with Li_2_O_2_ and promotes the electrolyte decomposition at high potentials (over 3.5 V), although carbon has been widely used as an air electrode matrix material because it has a large specific surface area and high electronic conductivity^31^,^32^,^33^,^34^. The high overpotential of Li-O_2_ cells for the oxygen evolution reaction is principally attributed to the non-conductive reaction products (such as Li_2_O_2_) deposited on the surface of air electrode. Furthermore, the unwanted reaction products require a high overpotential for the reverse reaction, i.e., to decompose (or else they easily accumulate on the surface of air electrode).

To address these issues in Li-O_2_ batteries, surface-modified carbon (carbon nanotubes, CNTs) and redox mediators were simultaneously introduced in the present work. In our previous studies, we demonstrated that modifying the surface of carbon with stable polymer coatings (polydopamine, polyimide, and poly(3,4-ethylenedioxythiophene) polystyrene sulfonate) effectively suppresses the side reactions on carbon-based air electrodes by limiting direct contact between the carbon and electrolyte and/or Li_2_O_2_ without significantly decreasing capacity^35^,^36^,^37^,^38^. Among the various polymer coating materials that we studied, a thin polyimide coating seemed to be the best approach to suppress side reactions at the air electrode, as demonstrated by the cycling performance of Li-O_2_ cells with the polymer-coated carbon electrodes^35^,^36^,^37^,^38^. However, the surface modification of carbon cannot decrease the high overpotential of Li-O_2_ cells upon charging. In this study, various redox mediators (namely, LiI, CsI, and LiNO_3_/CsI) were combined with the surface-coated carbon electrode to decrease the overpotential while maintaining excellent cycling performance. Redox mediators dissolved in the electrolyte can facilitate the oxidation of nonconductive reaction products. Redox mediators replace the slow reaction between the two solid phases (i.e., the reaction products and the air electrode) with a fast reaction between the liquid phase (i.e., the redox mediator dissolved in the electrolyte) and the solid reaction products, which significantly lowers the overpotential of the Li-O_2_ cells^39^,^40^,^41^,^42^,^43^,^44^,^45^,^46^,^47^,^48^,^49^. Therefore, Li-O_2_ cells fabricated from air electrodes based on polyimide-coated CNTs combined with a redox mediator in the electrolyte are expected to exhibit a low overpotential and excellent cycling performance due to the synergic effect of the two approaches.

## Results and Discussion

**Figure 1:**
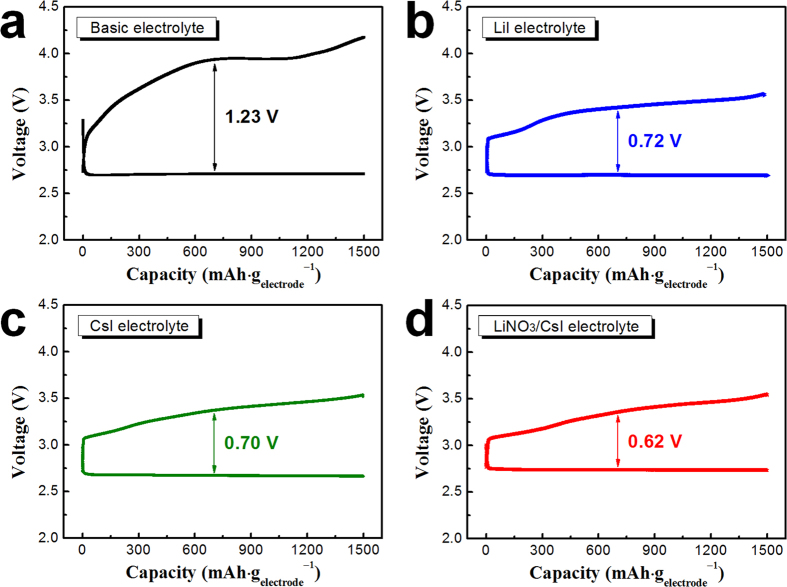
Initial discharge-charge profiles of the cells with polyimide-coated CNT electrodes using electrolytes with (**a**) basic; (**b**) LiI; (**c**) CsI; and (**d**) LiNO_3_/CsI (capacity was limited to 1,500 mAh·g_electrode_^−1^).

In order to characterize the effect of redox mediator, the electrochemical performance of the electrode composed of polyimide-coated CNTs was measured using a basic electrolyte and three electrolytes containing redox mediators (LiI, CsI and LiNO_3_/CsI). The basic electrolyte comprises 1 M lithium bis(trifluoromethanesulfonyl)imide (LiTFSI) in tetraethylene glycol dimethyl ether (TEGDME). Two of the experimental electrolytes contain either 0.05 M LiI or 0.05 M CsI added to the basic electrolyte (hereafter referred to as the LiI electrolyte and CsI electrolyte, respectively). The fourth electrolyte also contains 0.05 M CsI but with the addition of 0.5 M LiNO_3_ (as not only a redox mediator but also another salt) and only 0.5 M LiTFSI in TEGDME, i.e., the LiNO_3_/CsI electrolyte. Figure 1 shows the initial discharge-charge profile of the cells with polyimide-coated CNTs. The current density was 500 mA·g_electrode_^−1^, and the capacity of the cells was limited to 1,500 mAh·g_electrode_^−1^ in order to prevent a large depth-of-discharge^50^. The average voltage difference between the charging and discharging of the cell was ~1.23 V using the basic electrolyte. In contrast, that of the cell using electrolytes containing redox mediator was considerably decreased to ~0.72 (LiI electrolyte), ~0.70 (CsI electrolyte), and ~0.62 V (LiNO_3_/CsI electrolyte). The effect of the redox mediator was also observed in the discharge-charge profiles of the cells with a higher capacity limit (5,000 mAh·g_electrode_^−1^). As shown in Supplementary Fig. S1, the overpotential of the cells was distinctly decreased by the introduction of redox mediators. These results confirm that the redox mediators facilitate the dissociation of reaction products such as Li_2_O_2_ at a lower potential because they can easily transfer electrons from the electrode to the reaction products. The I^−^ ions in the LiI and CsI redox mediators are directly oxidized (I_3_^−^ or I_2_) at the surface of air electrode and are then reduced back to I^−^ upon dissociation of the reaction product (Li_2_O_2_)^42^,^43^,^44^,^45^,^46^,^47^,^48^,^49^. During that process, electrons from the Li_2_O_2_ easily transfer to the air electrode via I^−^ ions. This facile electron transfer lowers the amount of extra energy required to dissociate the reaction product and thus decreases the overpotential of the cells.

**Figure 2:**
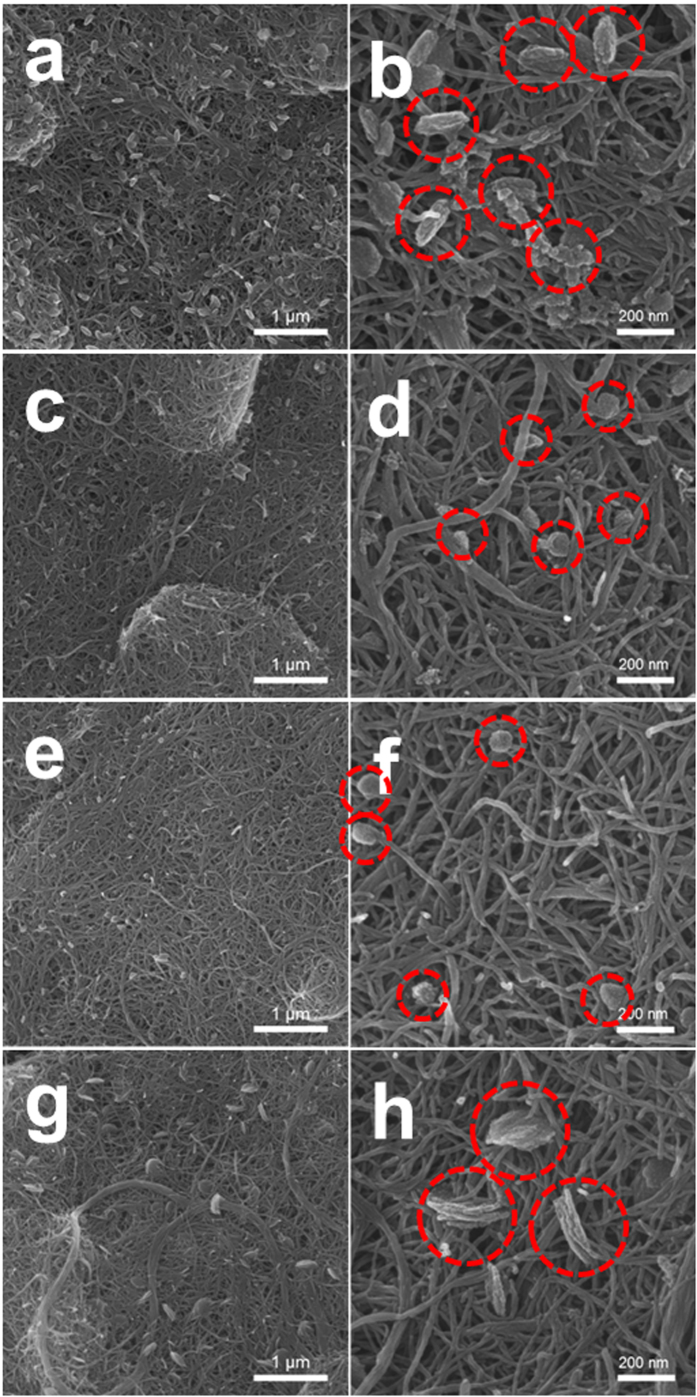
SEM images of the discharged electrodes employing polyimide-coated CNTs using electrolytes with (**a**,**b**) basic; (**c**,**d**) LiI; (**e**,**f**) CsI; and (**g**,**h**) LiNO_3_/CsI (capacity was limited to 1,500 mAh·g_electrode_^−1^).

Figure 2 shows the morphology of the electrodes after the initial discharge with a limited capacity of 1,500 mAh·g_electrode_^−1^. Typically, the reaction product forms as a film or particles on the surface of the electrode, and the morphology of the deposits is highly dependent upon the current density^8^ and solvent of the electrolyte^50^,^51^,^52^. However, the electrodes in the figures were tested using same current density (500 mA·g_electrode_^−1^) and solvent (TEGDME), i.e., the only differences between the cells were the electrolyte salts. Therefore, any changes observed in the surface morphology of the electrodes due to the formation of reaction products may be dependent upon the redox mediator (which may act as a salt) in the electrolytes. As shown in Fig. 2a and b, the surface of the discharged electrode using the basic electrolyte was covered with particle-type reaction products (marked with red circles) as well as a film-type reaction product coated on the surface of the CNTs. The surface of the electrodes using LiI and CsI electrolytes also exhibited particles, but they appeared smaller than those from the basic electrolyte (Fig. 2c–f). Interestingly, the reaction product particles grew larger when the LiNO_3_/CsI electrolyte was used, as shown in Fig. 2g and h. This morphological change was more distinct on the discharged electrode with the higher limited capacity (5,000 mAh·g_electrode_^−1^). As shown in Supplementary Fig. S2, a disc-shaped reaction product was dispersed on the surface of the discharged electrode using the basic electrolyte. The discharged electrode using the LiI and CsI electrolytes showed somewhat smaller reaction products. However, the discharged electrode using LiNO_3_/CsI electrolyte presented larger disc-shaped reaction products that those of the electrode using basic electrolyte, implying that LiNO_3_ may activate the growth of particle-type reaction products. Considering the morphological changes on the electrode surfaces, we cannot exclude the possibility that the reaction product itself can be changed by the redox mediator. To examine the reaction products, the electrodes were observed after the initial discharge using Fourier-transform infrared (FTIR) spectroscopy. As shown in Supplementary Fig. S3, the FTIR spectra of the electrodes using the four types of electrolytes seem to be very similar, confirming the presence of Li_2_O_2_ as a reaction product in each case.

**Figure 3:**
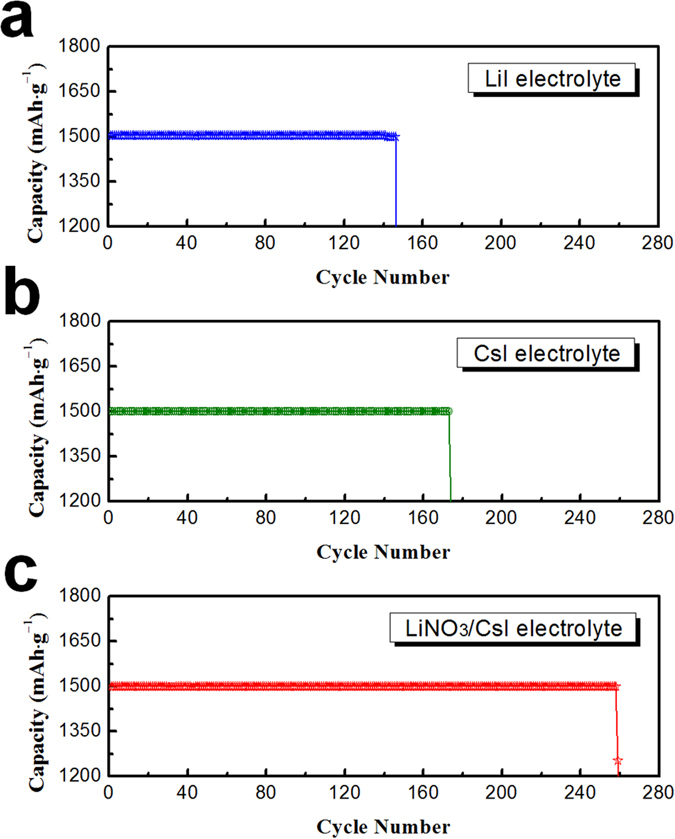
Cycling performance of the cells with polyimide-coated CNT electrodes using electrolytes with (**a**) LiI; (**b**) CsI; and (**c**) LiNO_3_/CsI (capacity was limited to 1,500 mAh·g_electrode_^−1^, and current density was 500 mA·g^−1^).

The cycling performance of the cells using electrolytes containing redox mediator was measured with a limited capacity of 1,500 mAh·g_electrode_^−1^. In our previous work^37^, cells employing polyimide-coated CNTs maintained their capacity (1,500 mAh·g_electrode_^−1^) for 137 cycles using the basic electrolyte, which is a much superior cycle life to that of the cells using the uncoated CNTs (65 cycles). This enhanced cycling performance is attributed to the polyimide coating layer on the surface of CNTs, which suppresses side reactions at the carbon/Li_2_O_2_ and carbon/electrolyte interfaces. As shown in Fig. 3a and b, the cells with polyimide-coated CNTs maintained their capacities for 146 and 173 cycles using LiI and CsI electrolytes, respectively, which are somewhat improved cycle lives compared to that measured with the basic electrolyte in our previous work (137 cycles). Notably, the cycling performance of the cells was dramatically enhanced using the LiNO_3_/CsI electrolyte. The cell with the LiNO_3_/CsI electrolyte and polyimide-coated CNTs maintained its capacity for 258 cycles, as shown in Fig. 3c. Considering that the limited capacity was 1,500 mAh·g_electrode_^−1^ (the limited capacity of the electrode for Li-O_2_ cells generally is under 1,000 mAh·g_electrode_^−1^), this is an exceptional cycle life for Li-O_2_ batteries. This result may be attributed to the synergic effect of the polyimide coating and the LiNO_3_/CsI electrolyte.

Generally, the cycle life of Li-O_2_ batteries is limited by several factors^17^,^18^,^19^,^20^,^21^. The accumulation of reaction products in the air electrode during cycling is one of the major factors limiting the cycle life. The insoluble reaction products form as a solid, which do not completely dissociate upon charging because of the slow kinetics between the solid reaction products and the solid air electrode. Moreover, side reactions produce unwanted reaction products such as Li_2_CO_3_ and organic materials (CH_3_CO_2_Li, HCO_2_Li, etc.), which barely dissociate during the charging process. The residual reaction products accumulate on the surface of the air electrode, resulting in capacity fading and a limited cycle life. The decomposition of the electrolyte is another factor limiting the cycling performance of Li-O_2_ cells. Basically, the instability of electrolyte is attributed to the inherent behaviour of Li-O_2_ cells, such as the formation of superoxide. However, this instability is also highly dependent upon the side reactions at the carbon/electrolyte interface. In conclusion, suppressing side reactions can stabilize the electrolyte and lessen the amount of residual reaction products on the air electrode. Considering that the side reactions are activated by carbon in the air electrode at high potentials (i.e., over 3.5 V)^31^,^32^,^33^,^34^, an effective approach for suppressing the side reactions may be both using the stable polyimide coating on the surface of the carbon (CNTs in the present study) and adding a redox mediator to the electrolyte, which lowers the overpotential.

**Figure 4:**
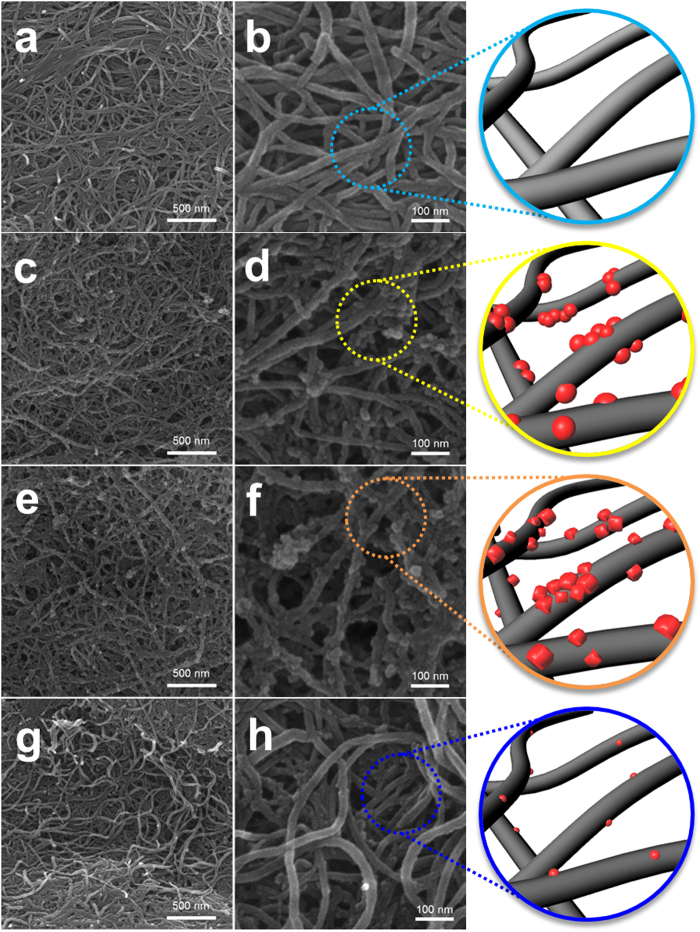
SEM images of the polyimide-coated CNT electrodes (**a**,**b**) before testing and after 50 cycles (charged state) using electrolytes with (**c**,**d**) LiI; (**e**,**f**) CsI; and (**g**,**h**) LiNO_3_/CsI.

In order to confirm the effects of our two-pronged approach, we analysed the air electrode after 50 cycles (charged state) using scanning electron microscopy (SEM) and FTIR spectroscopy. Figure 4 shows SEM images of the air electrodes composed of polyimide-coated CNTs obtained before the electrochemical test (pristine) and after 50 cycles (charged state) using the LiI, CsI, and LiNO_3_/CsI electrolytes. The pristine air electrode clearly exhibited the fibrous texture of the CNTs (Fig. 4a and b). The electrodes with uncoated and polyimide-coated CNTs were already characterized using the basic electrode in the previous work^37^. In that study, the uncoated CNT electrode was almost buried with the accumulated reaction products after 50 cycles, although the electrode was in the charged state. In contrast, the polyimide-coated CNT electrode showed fewer residual reaction products after cycling using the basic electrode because the polyimide coating suppressed the unwanted side reaction. However, the fibrous texture of the CNTs was still considerably covered with reaction products^37^. In this study, after cycling with the LiI and CsI electrolytes, the polyimide-coated CNT electrodes in Fig. 4c–f clearly showed the fibrous texture clearly and presented a porous structure with large holes and vacant spaces. This indicates that the redox mediators (LiI and CsI) contribute to diminishing the residual reaction products during cycling. However, the small particles were still observed on the surface of the CNTs, which are expected to be residual reaction products. In contrast, when the LiNO_3_/CsI electrolyte was used, the polyimide-coated CNTs maintained a relatively clear surface texture during cycling, as shown in Fig. 4g and h. The heterogeneous particles were almost absent compared to the electrodes cycled using LiI and CsI electrolytes. On the initially discharged electrode, we observed large reaction products, as shown in Fig. 2g and h. Therefore, the clear CNT surfaces of the cycled electrode indicate that the reaction products were effectively dissociated, and the unwanted side reactions were significantly suppressed using the LiNO_3_/CsI electrolyte.

**Figure 5:**
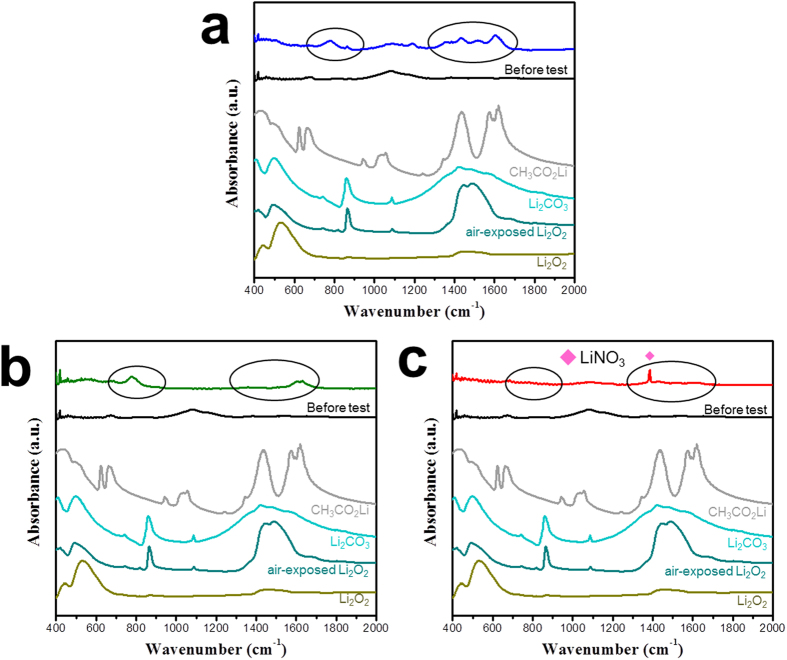
FTIR spectra of the polyimide-coated CNT electrodes after the 50^th^ cycle (charged state) using electrolytes with (**a**) LiI; (**b**) CsI; and (**c**) LiNO_3_/CsI.

Figure 5 presents the FTIR spectra of the electrodes recorded after 50 cycles (charged state) using the LiI, CsI, and LiNO_3_/CsI electrolytes. As shown in Fig. 5a, the electrode cycled using the LiI electrolyte exhibited broad peaks at 750–900, 1,350–1,500, and 1,500–1,700 cm^−1^, which are attributed to the unwanted reaction products such as Li_2_CO_3_ and CH_3_CO_2_Li. When the electrode was cycled using the CsI electrolyte, the peaks related to the unwanted reaction products remained, but they were somewhat decreased (Fig. 5b). However, in the electrode cycled with the LiNO_3_/CsI electrolyte, these peaks almost vanished, as shown in Fig. 5c. The SEM and FTIR spectra confirm that the redox mediator effectively decreased the residual reaction products and suppressed the side reaction during cycling. Specifically, the combination of CsI and LiNO_3_ was the best solution in our work. The side reactions activated at high potentials can be suppressed, because the decreased overpotential due to the redox mediator (as shown in Fig. 1) decreased the potential range during the charging process. Note that the overpotential of the cell containing redox mediators increased as the cycling proceeded (Fig. 4S). However, in the cells cycled using the LiNO_3_/CsI electrolyte, the overpotential increased much more slowly than in the cells cycled using the LiI and CsI electrolytes, as shown in Supplementary Fig. S4. This implies that the LiNO_3_/CsI electrolyte more effectively suppresses the side reactions than the other electrolytes, which results in the superior cyclic performance of the cell cycled using the LiNO_3_/CsI electrolyte. LiNO_3_ can stabilize the surface of carbon during the operation of the cells^49^, which may contribute to the slow increase in the overpotential and the decrease in the side reactions of Li-O_2_ cells during cycling.

**Figure 6:**
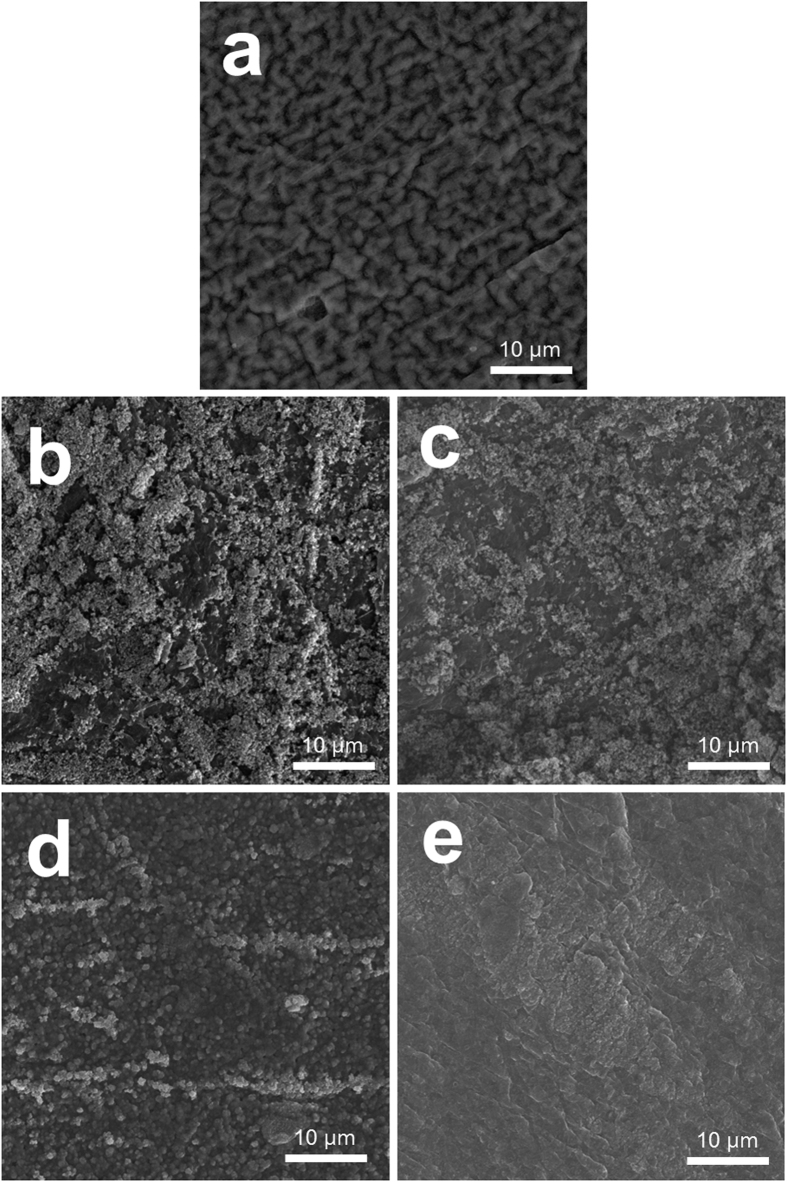
SEM images of the surface of the Li anode before testing (pristine) and after cycling. (**a**) Pristine and after cycling using the (**b**) basic; (**c**) LiI; (**d**) CsI; and (**e**) LiNO_3_/CsI electrolytes.

The instability of Li anodes during cycling, including the formation of dendrites, is another important factor limiting their cycling performance^53^,^54^,^55^. In order to examine the effect of LiI, CsI, and LiNO_3_/CsI electrolytes on the surface of the Li anode, the anodes were collected after 5 cycles and characterized using SEM and X-ray photoelectron spectroscopy (XPS) analyses. As shown in Fig. 6a, the surface of the Li anode before electrochemical test was smooth. In contrast, the Li anode after cycling using the basic electrolyte was rough and porous due to dendrite formation (Fig. 6b). These dendrites may grow during cycling and deteriorate the cell performance, including the cycle life. Clearly, dendrites formed on the surface of the Li anode cycled using the LiI electrolyte. However, the dendrite growth was significantly suppressed with the CsI electrolyte, which is attributed to the Cs^+^ ions. At a low concentration, Cs^+^ ions can interrupt the growth of dendrites because the Cs^+^ ions attached to the sharp points of the Li anode act as an electrostatic shield against additional Li growth^54^,^55^. As shown in Fig. 6e, the surface of the anode cycled using LiNO_3_/CsI electrolyte was very even and smooth, which implies that LiNO_3_ contributes to suppressing the Li dendrites. Although Cs^+^ ions and LiNO_3_ do not seem to completely eliminate dendrites over many cycles, they may delay the degradation of cell performance due to dendrite growth. Thus, this effect may explain the superior cycle life of the cells using CsI and LiNO_3_/CsI electrolytes compared to that of the cell using the LiI electrolyte.

**Figure 7:**
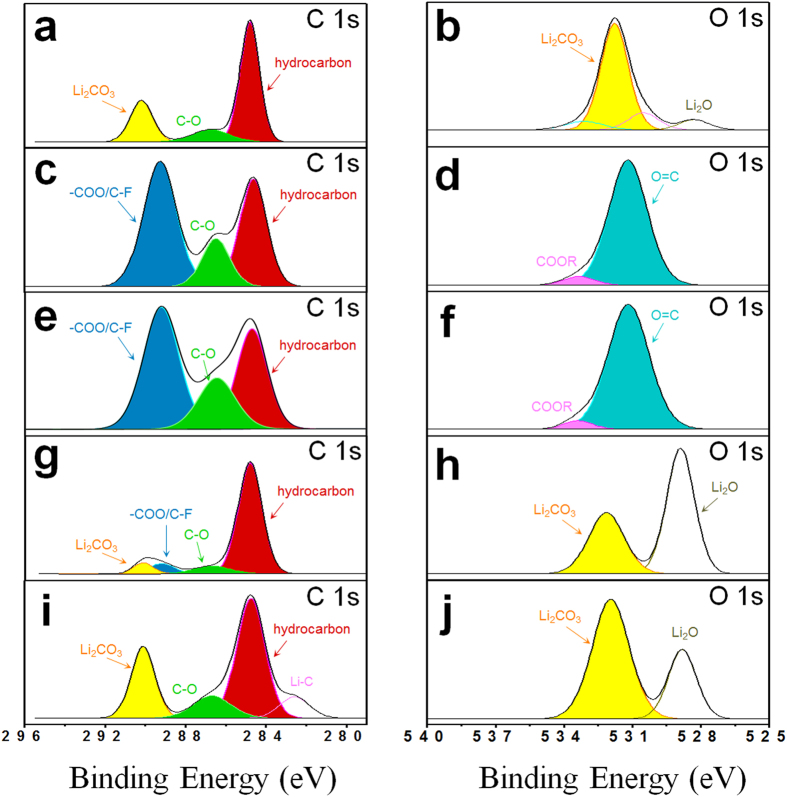
XPS spectra of C 1 s (left column) and O 1 s (right column) peaks for the Li anode before testing (pristine) and after five cycles. (**a**,**b**) Pristine and cycled using the (**c**,**d**) basic; (**e**,**f**) LiI; (**g**,**h**) CsI; and (**i**,**j**) LiNO_3_/CsI electrolytes.

Figure 7 presents the XPS spectra of C 1 s and O 1 s for the Li anode before and after five cycles using the basic, LiI, CsI, and LiNO_3_/CsI electrolytes. The C 1 s spectra of the Li anode before testing exhibited Li_2_CO_3_ (~290.1 eV) and hydrocarbon (~285 eV) peaks (Fig. 7a), which are attributed to the contamination of the Li surface during storage. In the O 1 s spectra of the Li anode before testing, Li_2_CO_3_ (~532 eV) and Li_2_O (~529 eV) peaks were detected, as shown in Fig. 7b. In contrast, the XPS spectra significantly changed after cycling. In the C 1 s spectra, the Li anode cycled using the basic and LiI electrolytes showed a large peak at ~298 eV corresponding to –COO and/or C-F (Fig. 7c and e). In addition, large O = C (~531.1 eV) and small COOR (~533.1 eV) peaks were observed in the O 1 s spectra (Fig. 7d and f). The formation of these chemical groups is attributed to side reactions such as electrolyte decomposition during cycling. Interestingly, in the C 1 s spectra of the Li anode cycled with the CsI electrolyte, the –COO and/or C-F peaks dramatically decreased and new Li_2_CO_3_ peaks appeared (Fig. 7g), which was observed in the spectra of the Li anode before testing. The Li anode cycled using the LiNO_3_/CsI electrolyte displayed C 1 s spectra similar to that of the Li anode before testing except for the small Li-C peak and a slight change in the intensities (Fig. 7i). Moreover, the O 1 s spectra of the Li anode cycled using the CsI and LiNO_3_/CsI electrolytes showed the same Li_2_CO_3_ (~532 eV) and Li_2_O (~529 eV) peaks as those of the Li anode before testing, although the Li_2_O peaks were more intense, as shown in Fig. 7h and j. The O = C (~531.1 eV) and COOR (~533.1 eV) peaks almost vanished using the CsI and LiNO_3_/CsI electrolyte. This XPS analysis also confirms that the CsI and LiNO_3_/CsI electrolytes can effectively suppress unwanted side reactions of Li-O_2_ cells. The Cs^+^ ions attached on the surface of the Li anode can decrease the side reactions at the highly vulnerable Li surface. The LiNO_3_ can be directly reduced to form surface Li_x_NO_y_ species^56^, thereby helping to stabilize the surface of the metallic Li anode. This synergic effect may contribute to the excellent cycling performance of the cell with the LiNO_3_/CsI electrolyte.

## Conclusion

**Figure 8:**
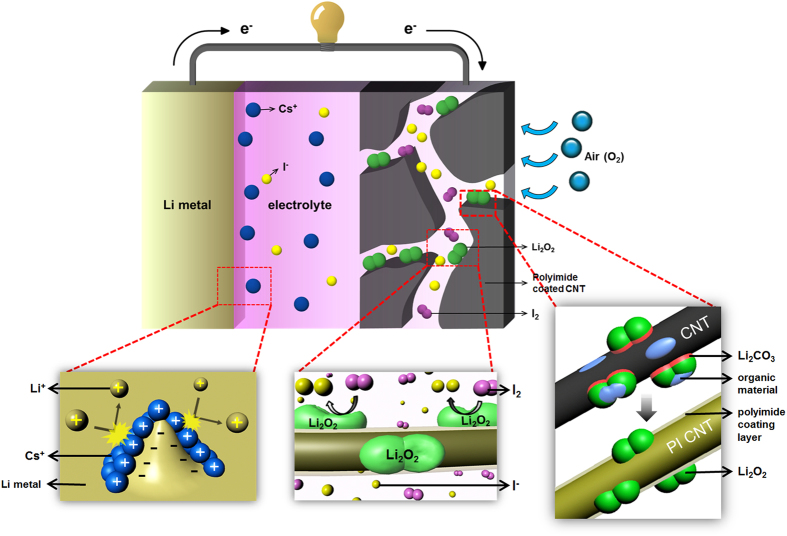
Schematic diagram showing the effect of polyimide-coated CNTs and the LiNO_3_/CsI electrolyte on the air electrode and anode (Li metal).

Air electrodes employing polyimide-coated CNTs were used in combination with electrolytes containing redox mediators (LiI, CsI, and LiNO_3_/CsI) for enhanced Li-O_2_ batteries. The introduction of the redox mediators effectively decreased the overpotential and improved the cycling performance of the polyimide-coated CNT electrodes. In particular, adding both CsI and LiNO_3_ to the electrolyte yielded the best results within our work. Figure 8 summarizes the effect of polyimide-coated CNTs and the LiNO_3_/CsI electrolyte on both the air electrode and anode (Li metal). In the air electrode, the polyimide coating on the surface of the CNTs can suppress the side reactions between the carbon and the Li_2_O_2_/electrolyte, thus decreasing the formation of unwanted reaction products such as Li_2_CO_3_ and organic materials (CH_3_CO_2_Li, HCO_2_Li, etc.). The I^−^ ions from CsI facilitate the dissociation of reaction products (Li_2_O_2_) and lower the overpotential. This lower overpotential can also suppress the side reactions that are activated at high potentials. In the Li anode, Cs^+^ ions from CsI interrupt the growth of dendrites via the electrostatic shield effect. LiNO_3_ may help to stabilize not only the carbon surface but also the Li anode. Because of the synergic effect of the coating and the redox mediators, cells fabricated from the polyimide-coated CNT electrode combined with the LiNO_3_/CsI electrolyte presented a low overpotential and an excellent cycle life.

## Methods

### Preparation of Polyimide-coated CNTs

The formation of the polyimide coating layer on the surface of commercial CNTs followed our previously reported method^37^. To form the polyimide coating on the CNTs surface, commercial CNTs were immersed in a 1.0 wt.% polyamic acid solution and then stirred for 30 min at room temperature. The CNTs were filtered from the solution by vacuum filtration and dried at 85 °C for 8 h. To convert the polyamic acid to polyimide, the polyamic-acid-coated CNTs were thermally treated in a nitrogen atmosphere via a multi-step imidization process (namely, 60 °C for 30 min → 120 °C for 30 min → 200 °C for 60 min → 300 °C for 60 min → 400 °C for 10 min).

### Preparation of air electrode

An air electrode was prepared by first mixing polyimide-coated CNTs (90 wt.%) with the polyvinylidene fluoride (PVDF) binder (10 wt.%) to obtain an electrode loading weight of 0.3 mg ± 0.03 mg.

### Electrochemical testing

The electrochemical performance of the electrodes was examined using a modified Swagelok cell consisting of an air electrode, a metallic Li anode, a Whatman glass filter separator, and an electrolyte. The cells were measured using four types of electrolytes, all dissolved in TEGDME: the ‘basic electrolyte’ with 1 M LiTFSI, the ‘LiI electrolyte’ with 0.05 M LiI and 1 M LiTFSI, the ‘CsI electrolyte’ with 0.05 M CsI and 1 M LiTFSI, and the ‘LiNO_3_/CsI electrolyte’ with 0.05 M CsI, 0.5 M LiNO_3_, and 0.5 M LiTFSI. The cells were assembled in an Ar-filled glove box and were subjected to galvanostatic cycling using a WonATech battery cycler (WBCs 3000). All experiments were conducted under an O_2_ atmosphere at ambient pressure.

### Characterization of the Electrode

FTIR (Nicolet 570) was measured for the electrodes to investigate the formation of Li_2_O_2_ and the amount of reaction products accumulated during cycling. SEM (Nova NanoSEM-450) was used to observe the surface morphology of the Li metal anode and the air electrodes. XPS (Thermo Scientific K-Alpha) was employed to analyse the surface reactions on the Li metal anode. To prepare specimens for SEM and XPS, the electrodes and Li metal anodes were separated from the cells, washed several times in dimethyl carbonate (DMC), and then stored in a vacuum chamber for 24 h. Furthermore, they were packed in a vacuum while being transferred to the instruments.

## Additional Information

**How to cite this article**: Yoon, S. H. and Park, Y. J. Polyimide-coated carbon electrodes combined with redox mediators for superior Li-O_2_ cells with excellent cycling performance and decreased overpotential. *Sci. Rep.* **7**, 42617; doi: 10.1038/srep42617 (2017).

**Publisher's note:** Springer Nature remains neutral with regard to jurisdictional claims in published maps and institutional affiliations.

## Supplementary Material

Supporting Information
