# Supplementary material for: Polyimide-coated carbon electrodes combined with redox mediators for superior Li-O2 cells with excellent cycling performance and decreased overpotential
Source: Sci Rep. 2017 Feb 15;7:42617. doi: 10.1038/srep42617 (PMC5309741; doi:10.1038/srep42617)
Supplement: Supporting Information [file srep42617-s1.pdf]

Supporting Information

Polyimide-coated carbon electrodes combined with redox mediators for superior Li-O_2 cells with excellent cycling performance and decreased overpotential

Seon Hye Yoon, Yong Joon Park *

Department of Advanced Materials Engineering, Kyonggi University, 154-42 Gwanggyosan-ro, Yeongtong-gu, Suwon-si, Gyeonggi-Do, 443-760, Korea

*Correspond to yjpark2006@kyonggi.ac.kr.

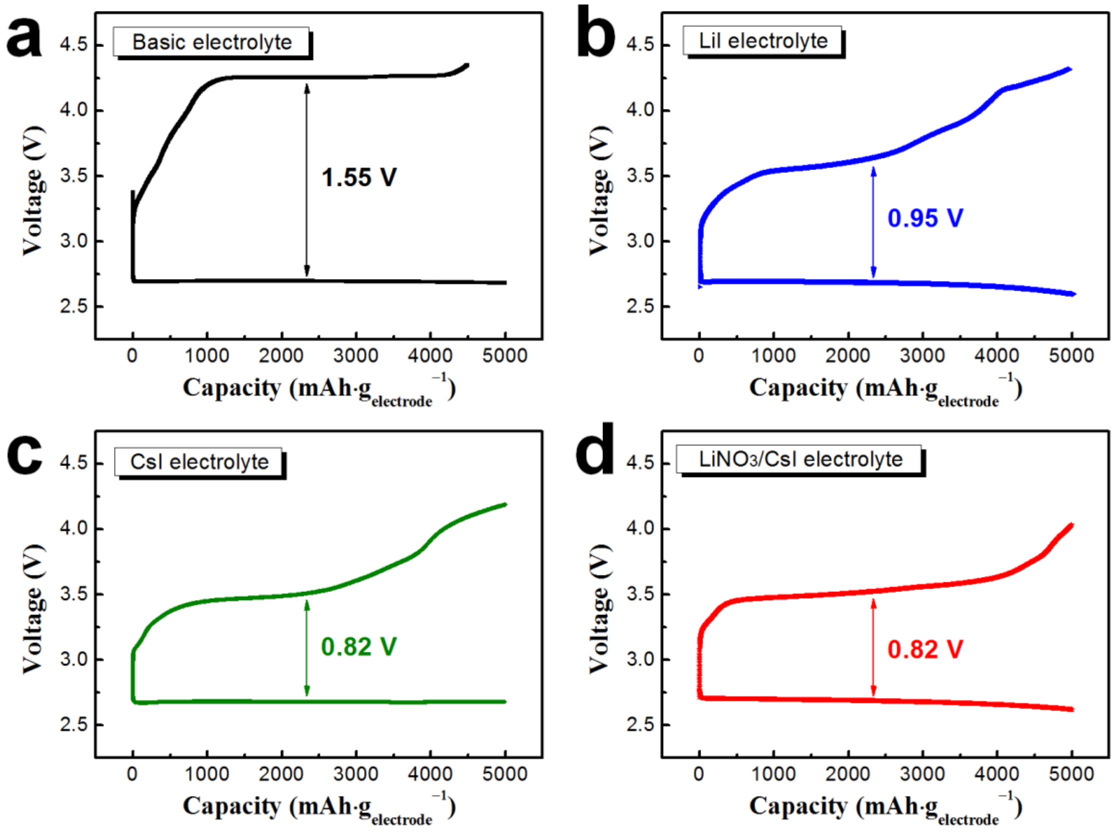

Figure S1. Initial discharge-charge profiles of polyimide-coated CNT electrodes using the (a) basic; (b) LiI; (c) CsI; and (d) $LiNO_3$/CsI electrolytes (capacity was limited to 5,000 $mAh \cdot g_{electrode}^{-1}$).

mAh·$g_{electrode}^{-1}$).

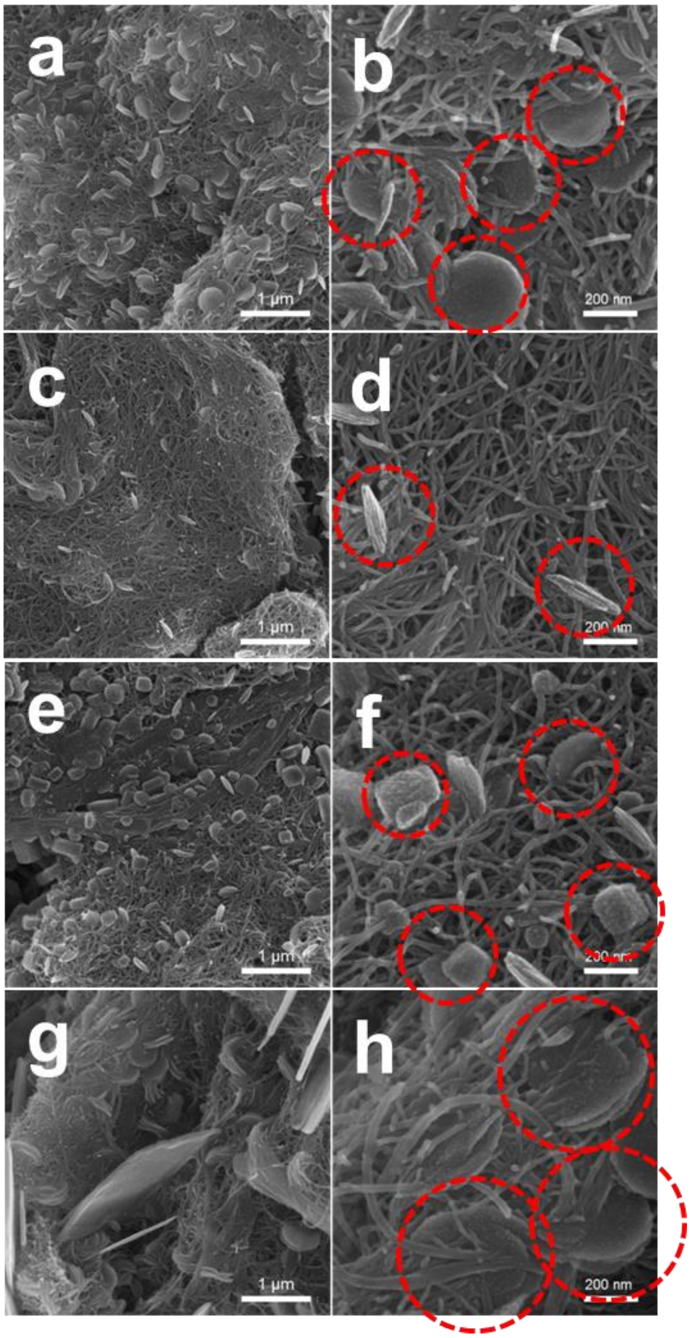

Figure S2. SEM images of the discharged polyimide-coated CNT electrodes using the (a, b) basic; (c, d) LiI; (e, f) CsI; and (g, h) $LiNO_3$/CsI electrolytes (capacity was limited to 5,000 mAh·$g_{electrode}^{-1}$).

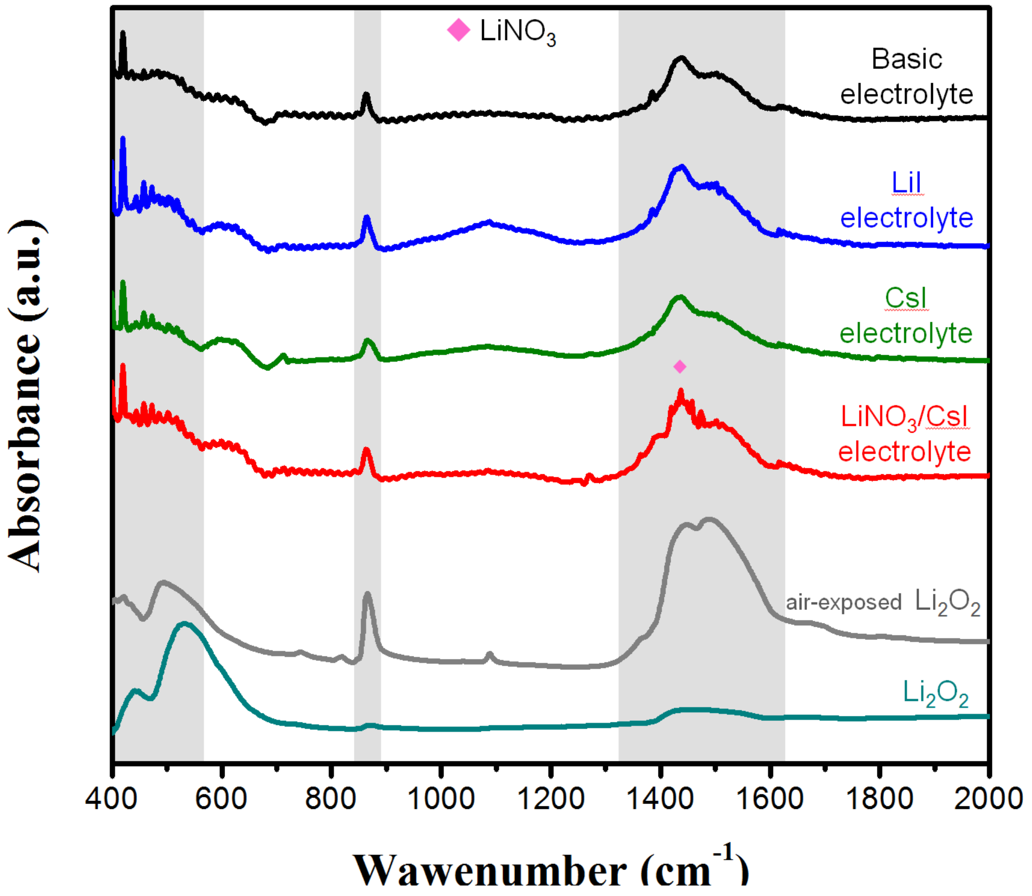

Figure S3. FTIR spectra of electrodes after the initial discharge process (capacity was limited to 5,000 mAh$\cdot g_{electrode}^{-1}$).

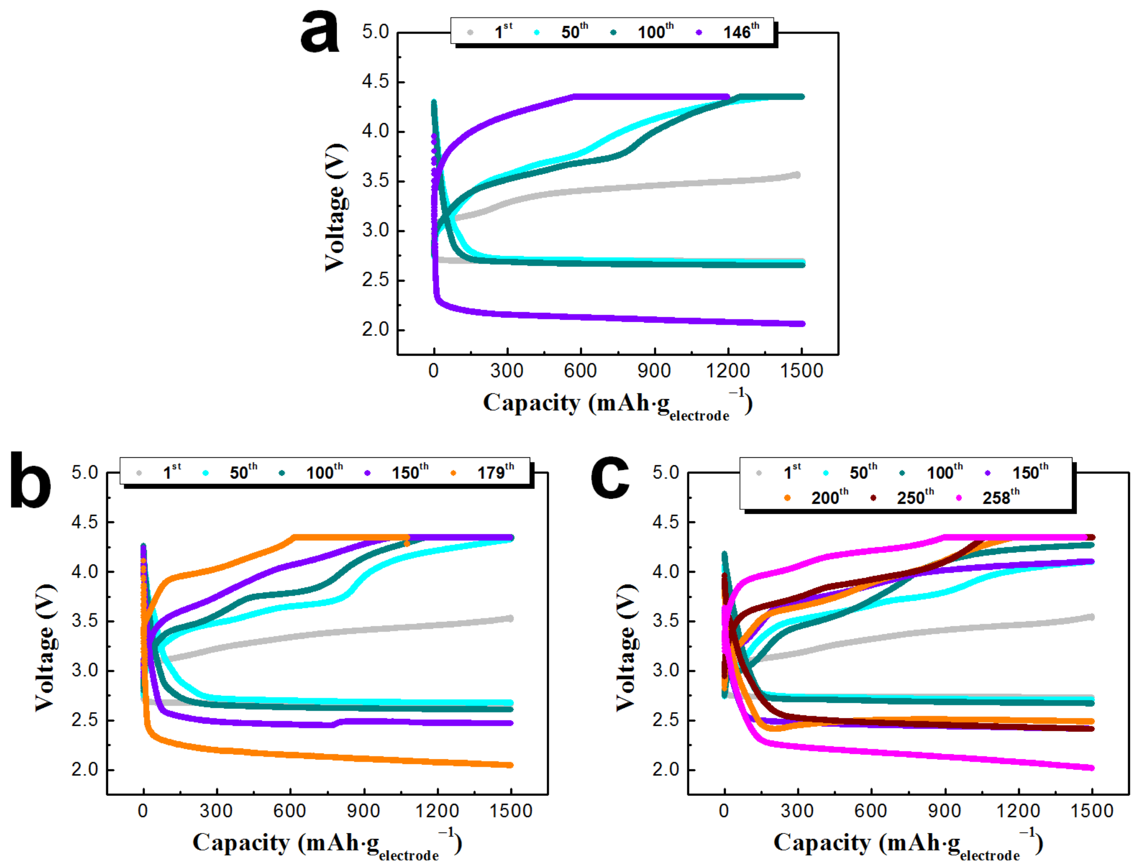

Figure S4. Discharge-charge profiles of the cells with polyimide-coated CNT electrodes using electrolytes with (a) LiI; (b) CsI; and (c) $LiNO_3$/CsI (capacity was limited to 1,500 mAh$\cdot g_{electrode}^{-1}$ and current density was 500 mA$\cdot g^{-1}$).
